# p38 mitogen-activated protein kinase is involved in arginase-II-mediated eNOS-Uncoupling in Obesity

**DOI:** 10.1186/s12933-014-0113-z

**Published:** 2014-07-18

**Authors:** Yi Yu, Angana G Rajapakse, Jean-Pierre Montani, Zhihong Yang, Xiu-Fen Ming

**Affiliations:** 1 Laboratory of Vascular Biology, Department of Medicine, Division of Physiology, University of Fribourg, Chemin du Musée 5, Fribourg, CH-1700, Switzerland

**Keywords:** Arginase II, eNOS-uncoupling, Obesity, p38mapk

## Abstract

**Background:**

Endothelial nitric oxide synthase (eNOS)-uncoupling links obesity-associated insulin resistance and type-II diabetes to the increased incidence of cardiovascular disease. Studies have indicated that increased arginase is involved in eNOS-uncoupling through competing with the substrate L-arginine. Given that arginase-II (Arg-II) exerts some of its biological functions through crosstalk with signal transduction pathways, and that p38 mitogen-activated protein kinase (p38mapk) is involved in eNOS-uncoupling, we investigated here whether p38mapk is involved in Arg-II-mediated eNOS-uncoupling in a high fat diet (HFD)-induced obesity mouse model.

**Methods:**

Obesity was induced in wild type (WT) and Arg-II-deficient (Arg-II^-/-^) mice on C57BL/6 J background by high-fat diet (HFD, 55% fat) for 14 weeks starting from age of 7 weeks. The entire aortas were isolated and subjected to 1) immunoblotting analysis of the protein level of eNOS, Arg-II and p38mapk activation; 2) arginase activity assay; 3) endothelium-dependent and independent vasomotor responses; 4) *en face* staining of superoxide anion and NO production with Dihydroethidium and 4,5-Diaminofluorescein Diacetate, respectively, to assess eNOS-uncoupling. To evaluate the role of p38mapk, isolated aortas were treated with p38mapk inhibitor SB203580 (10 μmol/L, 1 h) prior to the analysis. In addition, the role of p38mapk in Arg-II-induced eNOS-uncoupling was investigated in cultured human endothelial cells overexpressing Arg-II in the absence or presence of shRNA against p38mapk.

**Results:**

HFD enhanced Arg-II expression/activity and p38mapk activity, which was associated with eNOS-uncoupling as revealed by decreased NO and enhanced L-NAME-inhibitable superoxide in aortas of WT obese mice. In accordance, WT obese mice revealed decreased endothelium-dependent relaxations to acetylcholine despite of higher eNOS protein level, whereas Arg-II^-/-^ obese mice were protected from HFD-induced eNOS-uncoupling and endothelial dysfunction, which was associated with reduced p38mapk activation in aortas of the Arg-II^-/-^ obese mice. Moreover, overexpression of Arg-II in human endothelial cells caused eNOS-uncoupling and augmented p38mapk activation. The Arg-II-induced eNOS-uncoupling was prevented by silencing p38mapk. Furthermore, pharmacological inhibition of p38mapk recouples eNOS in isolated aortas from WT obese mice.

**Conclusions:**

Taking together, we demonstrate here for the first time that Arg-II causes eNOS-uncoupling through activation of p38 mapk in HFD-induced obesity.

## Background

Obesity and obesity-associated metabolic disorders are important risk factors of ischemic coronary heart disease [[1]]. Clinical and experimental studies demonstrate that obesity is associated with decreased endothelial nitric oxide (NO) bioavailability [[2],[3]], a hallmark of atherosclerotic vascular disease [[4]]. The endothelium-derived NO, produced from the substrate L-arginine by endothelial NO synthase (eNOS), exerts vascular protective effects through vasodilatation, inhibition of thrombosis, smooth muscle cell proliferation and vascular inflammation [[5]]. eNOS-uncoupling in which the enzyme generates superoxide anion instead of NO has been shown to be an important mechanism of endothelial dysfunction under numerous physiological and pathological conditions including aging, atherosclerosis, and obesity [[6]]. The mechanisms of eNOS-uncoupling are multifactorial and have not been fully elucidated [[7]]. Recent studies including our own demonstrate that the L-arginine ureahydrolase, arginase, including type-I and type-II arginase (Arg-I and Arg-II), is involved in eNOS-uncoupling in vascular diseases [[8]-[10]].

The endothelial cells express both Arg-I and Arg-II and share similar functions with respect to the negative regulation of eNOS functions [[9]]. Compelling evidence implicates that Arg-II plays a dominant role in eNOS-uncoupling in human and mouse blood vessels [[10],[11]]. Our recent studies demonstrate that genetic ablation of Arg-II in mice reduces atherosclerosis on the ApoE^-/-^ background, improves glucose homeostasis and insulin sensitivity in mice fed a high fat diet (HFD), which is at least partly attributable to dampening of macrophage inflammatory responses [[12]]. Moreover, genetic ablation of Arg-II slows down endothelial senescence and protects mice from age-associated endothelial inflammatory responses through inhibition of eNOS-uncoupling [[10]], implying a causative role of Arg-II in eNOS-uncoupling and endothelial dysfunction in aging. Although eNOS-uncoupling has been described in obesity [[13]], it is however not known whether Arg-II plays a role in this context in obesity.

Evidence has been presented that p38mapk is an important signaling pathway sensing cellular stress and is involved in pathogenesis of cardiovascular diseases and eNOS-uncoupling [[14],[15]]. Mammalian p38mapk has four isoforms, α, β, δ, γ, which are expressed to different extent in specific cells and tissues, and function as sensors to various stressors, including oxidative stress, inflammatory stimuli, oncogenes [[14],[16]]. Of the four enzyme isoforms, p38α is the best recognized isoenzyme of cardiovascular importance [[14]]. Pharmacological inhibition of p38mapk has been shown to reduce atherogenesis and improve plaque stability in animal models [[17],[18]] and protects against ischemic myocardial injury [[14],[19]], which can be partly attributed to inhibition of inflammatory responses and foam cell formation [[20],[21]]. In endothelial cells, p38mapk has been reported to mediate apoptosis and senescence [[22],[23]]. Inhibition of p38mapk in animals and humans is able to improve endothelium-mediated vascular relaxations [[15],[20]], and p38mapk has been shown to be increased in the arteries of obesity animal models [[24]], suggesting a role of p38mapk in endothelial dysfunction in obesity.

Previous studies suggest that p38mapk acts upstream of Arg-II, which mediates endothelial dysfunction of corpus cavernosum in angiotensin II-induced hypertension mouse model [[25]]. However, the effects of p38mapk and Arg-II on endothelial dysfunction in obesity and insulin resistance are not known. The aim of our current study is to investigate whether Arg-II plays a role in eNOS-uncoupling in obesity, and whether p38mapk is involved.

## Materials and methods

### Materials

Reagents were purchased or obtained from the following sources: rabbit antibody against Arg-II was from Santa Cruz Technology Inc. (Nunningen, Switzerland); mouse antibodies against eNOS and p38mapk, rabbit antibodies against phospho-p38mapk (Thr180/Tyr182) and phospho-Ser1177-eNOS were purchased from Cell Signaling (Allschwil, Switzerland); mouse antibody against tubulin was from Sigma (Buchs, Switzerland). IRDye 800-conjugated affinity purified goat anti-rabbit IgG F(c) was purchased from BioConcept (Alschwil, Switzerland), Alexa fluor 680-conjugated goat anti–mouse IgG (H + L) was from Invitrogen (Lucerne, Switzerland). Dihydroethidium (DHE) was from Molecular Probes/Invitrogen (Lucerne, Switzerland), and the membrane-permeable 4,5-diaminofluoresceine diacetate (DAF-2DA) was from VWR international SA (Dietikon, Switzerland). L-norepinephrine bitartrate, acetylcholine (ACh), sodium nitroprusside (SNP) and SB203580 were purchased from Calbiochem. All cell culture media and materials were purchased from Gibco BRL (Lucerne, Switzerland).

### Animals

The Arg-II^-/-^ mice were kindly provided by Dr. William O’Brien [[26]] and backcrossed to C57BL/6 J for more than ten generations [[12]]. Genotyping was performed by polymerase chain reaction (PCR) as previously described [[26]]. The WT and Arg-II^-/-^ offsprings from hetero/hetero cross were interbred to obtain WT and Arg-II^-/-^ mice, respectively, for experiments. Starting at the age of 7 weeks, the male WT and Arg-II^-/-^ mice were given free access during 14 weeks to either a normal chow (NC; energy content: 10.6% fat, 27.6% protein, and 57% carbohydrate, fiber 4.8%; Provimi Kliba NAFAG 3436; Kaiseraugst, Switzerland) or a high fat diet (HFD, energy content: 55% fat, 21% protein, and 24% carbohydrate; Harlan Teklad TD 93075; Horst, Netherlands). Animals were sacrificed after 14 weeks of HFD. The entire aortas from the heart to the iliac bifurcation were removed, placed into cold (4°C) Krebs bicarbonate solution, dissected free from fat and adhering perivascular tissue. The isolated aortic rings were used either for vasomotor response measurement in an organ chamber setup (see below), *en face* staining of Superoxide anion and NO (see below), or snap-frozen in liquid nitrogen and kept at -80°C until used for immunoblotting analysis and arginase activity assay. Animal handling and experimentation were approved by the Service de la sécurité alimentaire et des affaires vétérinaires, Etat de Fribourg.

### Generation of recombinant adenovirus (rAd)

Generation of rAd expressing shRNA targeting human p38mapkα driven by the U6 promoter (rAd/U6-hp38α^shRNA^) was carried out with the Gateway Technology (Invitrogen Life Technologies) according to manufacturer’s instructions. The targeting sequence for hp38α-shRNA is indicated in boldface below (only the sense strand is shown):

 5′-CACC**GTTACGTGTGGCAGTGAAGAA**CGAATTCTTCACTGCCACACGTAAC-3′.

rAd/U6-LacZ^shRNA^, rAd/CMV empty vector and rAd/CMV-Arg-II were generated as previously described [[10]].

### Endothelial cell culture and adenoviral transduction of the cells

Cultivation and transduction of Human umbilical vein endothelial cells (HUVECs) were performed as previously described [[11]]. Cells were transduced with the rAd at titers of ~200 multiplicities of infection and then cultured in complete medium for 2 to 4 days before experiments.

### Detection of NO and superoxide level in cultured endothelial cells and in intact mouse aortas

NO and superoxide levels in cultured endothelial cells as well as in intact mouse aortas were assessed by staining the cells or aortas *en face* with fluorescent dyes DAF-2DA and DHE, respectively, as described previously [[27]]. Briefly, Z-scanning was done for each sample. After the signal on the top (endothelial layer on the lumen border) of the sample was observed, the images were collected. Three consecutive images per field, acquired through the full thickness of endothelial signal, were recorded for analysis. At least 3 different fields per sample were evaluated. The images from DAF-2DA and DAPI staining were quantified with Image J software and results are presented as the ratio of DAF-2DA and DAPI or ratio of DHE and DAPI positive nucleus.

### Measurement of arginase activity

Arginase activity in the aortic tissue lysates was measured by colorimetric determination of urea formed from L-arginine in an in vitro activity assay as previously described [[11]].

### Immunoblotting analysis

Preparation of mouse aortic tissue and endothelium cell extract, SDS-PAGE, transfer of SDS gels to an Immobilon-P membrane (Millipore) were performed as previously described [[12]]. The resultant membrane was incubated overnight with the corresponding primary antibody (1:2500 for eNOS, 1:500 for phospho-Ser1177-eNOS, 1:200 for arginase-II and 1:1000 for p38mapk and phospho-Thr180/Tyr182-p38mapk) at 4°C with gentle agitation after blocking with 5% skimmed milk. The protein was decorated with a corresponding anti-mouse (Alexa fluor 680 conjugated) or anti-rabbit (IRDye 800 conjugated) and detected by Odyssey Infrared Imaging System (LI-COR Biosciences). Quantification of the signals was performed using the Odyssey Application Software 1.2.

### Endothelium-dependent and independent responses

Endothelium-dependent and independent relaxations were studied as previously described [[11]]. Briefly, the descending thoracic aortas with intact endothelium cleaned of perivascular tissues were cut into rings (3 mm in length) and then suspended in a Multi-Myograph System (Model 610 M, Danish Myo Technology A/S, Denmark). The endothelium-dependent relaxations in response to acetylcholine (1 nmol/L to 10 μmol/L) and endothelium-independent relaxations in response to the NO donor sodium nitroprusside (SNP, 0.1 nmol/L to 1 μmol/L) were then performed in aortic rings precontracted with norepinephrine (0.1 to 0.3 μmol/L) to match the precontraction.

### Statistics

Data are given as mean ± SEM. In all experiments, n indicates the number of individual animals used or of individual experiments when conducted with cultured cells. Statistical analysis was performed with unpaired Student *t* test or ANOVA with Dunnett or Bonferroni post-test. Differences in mean values were considered significant at p < 0.05.

## Results

### Augmented Arg-II expression/activity and p38mapk activation in the aortas of obese mice

**Figure 1:**
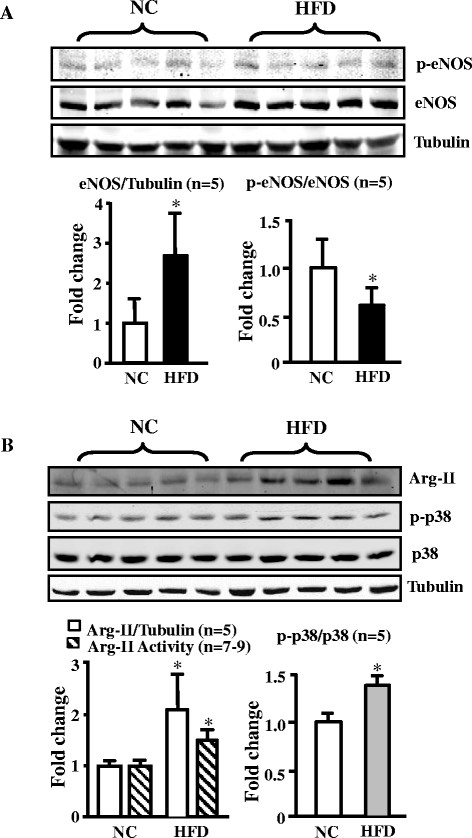
**HFD feeding enhances eNOS, Arg-II expression/activity, and p38mapk activation in mouse aortas. (A)** Immunoblotting analysis of eNOS total protein and eNOS-S1177 (p-eNOS) levels (n = 5). **(B)** Immunoblotting analysis of Arg-II (Arg-I not detectable, tubulin served as loading control), p38mapk activation, i.e., Thr180/Tyr182-phosphorylated p38mapk (p-p38mapk) and total p38mapk in the aortas of WT mice fed NC or HFD (n = 5), and arginase activity assay (n = 7-9). *p < 0.05 vs NC. In case of analyzing total protein levels such as Arg-II and eNOS, the ratio of Arg-II/tubulin or eNOS/tubulin in NC group serves as reference. The levels of eNOS and Arg-II expression in HFD group are calculated as fold changes to those of the NC group.

To study the role of Arg-II in eNOS dysfunction in obesity, eNOS levels and Arg-II expression/activity in the aortas of mice fed HFD were analyzed. Our previous study showed that Arg-II^-/-^ mice are protected from low grade systemic inflammation, insulin resistance, and glucose intolerance, despite comparable body weight under HFD feeding [[12]]. In this study, we further analyzed vascular endothelial function in the obesity mouse model and showed that the protein level of eNOS in the aortas of obese mice fed HFD was significantly higher than that of lean mice fed NC, while the activating eNOS-S1177 phosphorylation level was comparable between the two groups, which results in a decreased eNOS-S1177/total eNOS ratio (Figure 1A). The results implicate a decreased activation of eNOS in HFD-induced obesity. In addition, a significantly higher Arg-II expression and enzymatic activity, and a small but significantly augmented p38mapk activation in the aortas were also observed in the obese mice as compared to the lean mice (Figure 1B), while Arg-I was below the detectable limit. The results suggest that increased Arg-II and p38mapk activation play important roles in endothelial dysfunction in obesity.

### eNOS-uncoupling in obesity

**Figure 2:**
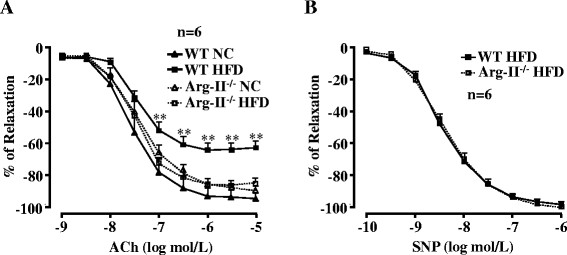
**Arg-II gene deficiency prevents HFD-induced impairment of endothelium-dependent relaxation. (A)** Endothelium-dependent relaxations in response to acetylcholine (ACh) were significantly impaired by HFD feeding in WT mice () and preserved in Arg-II^-/-^ mice (). Arg-II deficiency had no significant effects in mice fed NC. **(B)** Arg-II deficiency does not affect endothelium-independent relaxations in response to the NO donor sodium nitroprusside (SNP) in animals fed HFD. n = 6, **p < 0.01 between WT HFD and Arg-II^-/-^ HFD groups.

**Figure 3:**
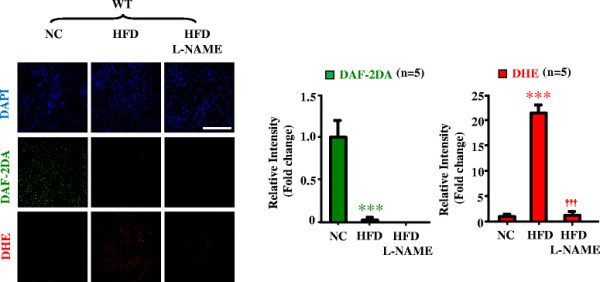
**eNOS-uncoupling in obesity.** Confocal microscopic *en face* detection of NO and O_2_^-^ by DAF-2DA and DHE staining, respectively, followed by counterstaining with DAPI of aortas. Aortas of WT mice fed NC or HFD were treated with or without the eNOS inhibitor L-NAME (1 mmol/L) for 1 hour followed by acetylcholine (ACh) stimulation (1 μmol/L, 10 minutes) and then DAF-2DA and DHE staining. n = 5; ***p < 0.005 vs NC; ^†††^p < 0.005 vs HFD group. Scale bar = 0.1 mm.

Next we examined whether enhanced Arg-II contributes to eNOS dysfunction in obesity. The endothelium-dependent relaxations in response to acetylcholine (ACh) were significantly impaired in WT obese mice fed HFD (Figure 2A). Although Arg-II deficiency did not significantly affect HFD-induced weight gain [[12]], it preserved the endothelium-dependent relaxations to ACh (Figure 2A), whereas the endothelium-independent relaxations in response to the NO donor sodium nitroprusside (SNP) under HFD feeding were not affected (Figure 2B). It is to note that Arg-II deficiency had no significant effects on endothelial function in lean mice fed NC (Figure 2A). The results demonstrate impairment of endothelial function not smooth muscle relaxation in obesity involving Arg-II. In accordance with this result, confocal fluorescence microscopy revealed a decrease in NO (DAF-2DA staining) and an increase in L-NAME-sensitive superoxide generation (DHE staining) in the aortic endothelial layer of WT mice fed HFD as compared to that of the WT mice fed NC (Figure 3), suggesting a role of Arg-II in eNOS-uncoupling in obesity.

### Arg-II mediates eNOS-uncoupling in obesity through p38mapk

**Figure 4:**
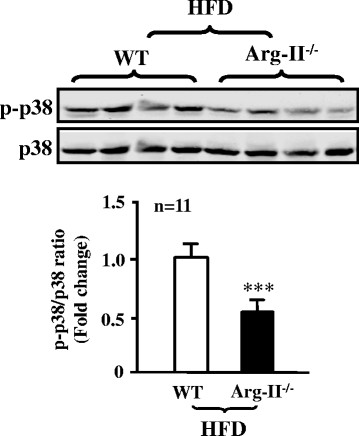
**Decreased p38mapk activation in aortas of obese Arg-II**^**-/-**^**mouse.** Immunoblotting analysis of the Thr180/Tyr182 phosphorylated p38mapk (p-p38mapk) and total p38mapk in aortas of WT and Arg-II^-/-^ mice fed HFD. n = 11, ***p < 0.005 vs WT.

**Figure 5:**
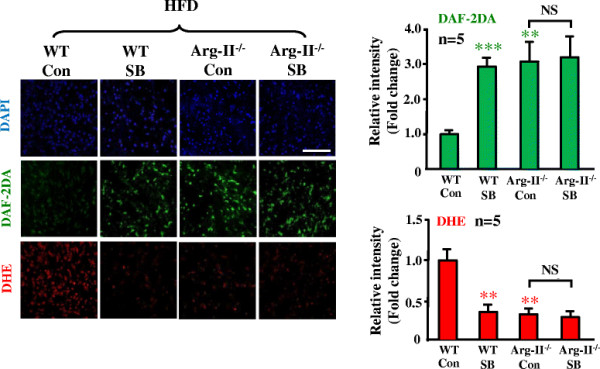
**Arg-II gene deficiency and inhibition of p38mapk prevent eNOS-uncoupling in obesity.** Confocal microscopic *en face* detection of NO with DAF-2DA and superoxide with DHE followed by counterstaining with DAPI in intact mouse aortas. Aortas from WT and Arg-II^-/-^ mice fed HFD were cleaned of perivascular tissues, cut into two parts and subjected to treatment with either DMSO as control (Con) or SB203580 (SB, 10 μmol/L) for 1 hour followed by treatment with acetylcholine (ACh, 1 μmol/L) for 10 minutes before DAF-2DA and DHE staining. n = 5, **p < 0.01 and ***p < 0.005 vs WT/Con group. Scale bar = 0.1 mm.

To study the role of p38mapk in Arg-II-mediated eNOS-uncoupling in obesity, we examined p38mapk activation in the aortas of the obese mice. As shown in Figure 4, activation of p38mapk as measured by phosphorylation levels of p38mapk at Thr180/Tyr182, which was detected in the aortas of WT mice fed HFD (Figure 1), was significantly decreased when Arg-II gene was deficient, implicating an interaction between Arg-II and p38mapk. To further demonstrate the role of p38mapk in Arg-II-mediated eNOS-uncoupling in obesity, aortas of WT mice fed HFD were treated with the p38mapk inhibitor SB203580 (10 μmol/L, 1 hour). The results showed that under the stimulation with ACh (1 μmol/L, 10 minutes), levels of NO production (DAF-2DA staining) in the intact endothelial layer of the aortas from WT mice fed HFD were significantly enhanced by the p38mapk inhibitor SB203580 (Figure 5). Importantly, NO production in Arg-II^-/-^ mice under HFD feeding was significantly higher than that in WT mice under HFD feeding, but comparable to the levels in the obese WT mice treated with SB203580. This preserved endothelial NO production in Arg-II^-/-^ mice fed HFD could not be further enhanced by the p38mapk inhibitor SB203580 (Figure 5), Moreover, the levels of superoxide generation (DHE staining) in the WT mice fed HFD were higher than Arg-II^-/-^mice, and this enhanced superoxide generation in obese WT mice was abolished by the p38mapk inhibitor to the levels as in the Arg-II^-/-^ mice (Figure 5). The results suggest a common mechanism of endothelial dysfunction mediated by Arg-II and p38mapk in obesity. The fact that the higher endothelial NO production and lower superoxide generation in the Arg-II^-/-^ mouse aortas could not be further affected by SB203580, demonstrates that Arg-II plays a causative role in eNOS-uncoupling in obesity through activation of p38mapk.

**Figure 6:**
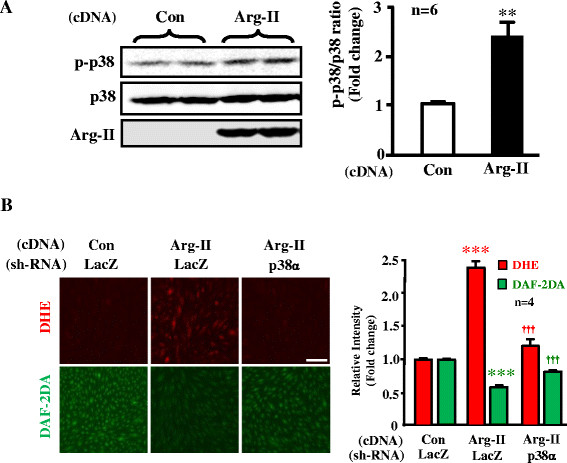
**p38mapk is involved in Arg-II-induced eNOS-uncoupling. (A)** In cultured HUVECs, overexpressing Arg-II gene led to elevated p38mapk activation as measured by the Thr180/Tyr182 phosphorylated p38mapk (p-p38mapk). n = 6, **p < 0.01 vs Con group. **(B)** HUVECs were transduced first with rAd/U6-LacZ^shRNA^ as control or rAd/U6-p38^shRNA^. Twelve hours after the 1^st^ transduction with the rAd/U6-shRNAs, the cells were then transduced either with rAd/CMV as control (Con) or with rAd/CMV-Arg-II to overexpress Arg-II (Arg-II). Experiments were performed 60 hours post the 2^nd^ transduction (48 hours in 5% FCS-RPMI-1640 medium plus overnight serum-starvation in 0.2% FCS-RPMI-1640). Shown are representative images of four independent experiments. ***p < 0.005 vs Con/LacZ group, ^†††^p < 0.005 vs Arg-II/LacZ group. Scale bar = 0.2 mm.

Moreover, experiments in cultured human endothelial cells showed that overexpression of Arg-II gene led to elevated p38mapk activation (Figure 6A), caused eNOS-uncoupling as revealed by enhanced superoxide generation (DHE staining) and reduced NO production (DAF-2DA staining), which was recoupled by silencing p38α, the major isoform of the enzyme in endothelial cells (Figure 6B). The results further strengthen the above conclusion that Arg-II causes eNOS-uncoupling involving p38mapk.

## Discussion

Endothelial dysfunction occurs in obesity and diabetes mellitus including type-I and type-II diabetes and is viewed as one of the most important mechanisms linking to diabetic cardiovascular complications [[28]]. Although multiple mechanisms underlying endothelial dysfunction in obesity and diabetes are demonstrated [[29]], functional defect of eNOS such as eNOS-uncoupling rather than decreased eNOS gene expression seems to be the major contributor to endothelial dysfunction in obesity and diabetes [[13]]. In line with this study, our present study demonstrates that despite augmented eNOS expression in the aortas of the obese WT mice (Figure 1), the endothelium-dependent relaxation (not the endothelium-independent relaxation) is decreased as compared to the lean mice fed NC (Figure 2), suggesting dysfunctional eNOS or uncoupling of eNOS in obesity. This conclusion is confirmed by the fact that in the obese WT mice, the increased superoxide generation is abolished by the eNOS inhibitor L-NAME (Figure 3). The enhanced eNOS expression in obesity is not clear and might reflect a compensatory mechanism to counteract oxidative stress under this condition.

Obesity is associated with systemic and vascular insulin resistance [[1]]. In the vascular endothelial cells, insulin is able to enhance eNOS activity through protein kinase B, PKB/Akt, which phosphorylates and activates eNOS at serine-1177 [[30],[31]]. Defects of insulin signaling in obesity therefore result in insulin resistance, glucose intolerance, and decreased vascular endothelial function [[31]]. Although our present study shows comparable eNOS-S1177 levels between lean and obese mice, we could demonstrate a decreased eNOS-S1177/eNOS ratio resulting from enhanced eNOS total protein levels in obesity (Figure 1), which implicates vascular insulin resistance in obesity. This mechanism, in addition to eNOS-uncoupling, must be involved in endothelial dysfunction in our mouse model.

In the present study, we further demonstrate that obesity-associated eNOS-uncoupling is due to enhanced Arg-II expression and activity. First, Arg-II expression and activity is augmented in the aortas of obese mice fed HFD (Figure 1); Second, genetic ablation of Arg-II fully preserved the endothelium-dependent relaxation in HFD-induced obesity (Figure 2). This protective effect of Arg-II gene deficiency on endothelium-dependent relaxation is attributable to recoupling of eNOS function, since HFD induced eNOS-uncoupling in WT mice as evidenced by decreased NO production and increased L-NAME-sensitive superoxide production in the aortic endothelial cells of the WT obese mice (Figure 3), while the aortic endothelial cells of Arg-II^-/-^ obese mice revealed increased NO and decreased superoxide production (Figure 5). These findings are in agreement with our recent studies demonstrating eNOS-uncoupling induced by Arg-II in aging animal models [[10]]. We have now extended this function of Arg-II to the obesity-associated type-II diabetes model. The role of Arg-II in decreasing eNOS function has also been reported by other studies showing that Arg-II activity and expression are enhanced in human diabetic corpus cavernosum, and inhibition of the enzyme enhances NO-dependent relaxations of corpus cavernosum smooth muscle in vitro [[25],[32],[33]].

Accumulating evidence indicates that p38mapk is involved in endothelial dysfunction and senescence [[23],[25]]. Studies demonstrate that p38mapk is activated in the aortas of angiotensin-II-induced hypertension and in obesity [[24],[34]]. In line with these studies, we also show significantly augmented p38mapk activation in the mouse aortas of HFD-induced obesity (Figure 1). Activation of p38mapk involves multiple mechanisms which are not fully understood, yet [[14]]. Hyperglycemia has been implicated in activation of p38mapk in various types of cells including endothelial cells, cardiomyoblasts, and dendritic cells [[35]-[37]]. This effect of glucose on p38mapk activation in endothelial cells is concentration- and time-dependent [[37],[38]]. In our obesity mouse model, increase in fasting plasma glucose concentration, glucose intolerance, and insulin intolerance were demonstrated [[12]]. Thus, hyperglycemia in our obesity mouse model could contribute to p38mapk activation in the vasculature. Besides the role of p38mapk in endothelial dysfunction, it has also been shown that p38mapk activation is the initial signaling event in the regulation of scavenger receptor expression by high glucose in human dendritic cells, that is required for subsequent activation of NF-κΒ [[36]]. These studies support the current concept that targeting p38mapk may affect functions of various cell types, leading to beneficial therapeutic effects in treatment of cardiovascular disease or dysfunctions associated with risk factors [[14]]. Indeed, the results from our present study demonstrate that pharmacological inhibition of p38mapk improves endothelial function by inhibition of eNOS-uncoupling in obesity (Figure 5).

The relationship of p38mapk and Arg-II has been reported in the literature. Inhibition of p38mapk has been shown to reduce arginase activity and Arg-II expression and improve corpus cavernosum tissue relaxation in these animal models [[25]]. These results indicate that p38mapk is the upstream signal that enhances Arg-II expression. In our current study, we provide for the first time the evidence showing that p38mapk acts as down-stream effector of Arg-II and mediates Arg-II-induced eNOS-uncoupling in cultured cells as well as in the obesity mouse model. This conclusion is supported by the following evidences. Firstly, overexpression of Arg-II in cultured human endothelial cells causes eNOS-uncoupling, which is prevented by silencing p38mapk (Figure 6); secondly, p38mapk activation is significantly reduced in the aortas of obese Arg-II^-/-^ mice as compared to the control WT littermates fed HFD (Figure 4); finally, treatment of the intact aortas with p38mapk inhibitor recouples eNOS function, i.e., causes inhibition of superoxide generation and enhances NO production in obese WT mice without any further effects on NO or superoxide level in obese Arg-II^-/-^ mice (Figure 5). It remains to be investigated how Arg-II activates p38mapk.

In conclusion, our study demonstrates that Arg-II causes eNOS-uncoupling through activation of p38mapk pathway in HFD-induced obesity. Targeting vascular Arg-II and/or p38mapk may represent a novel therapeutic approach for treatment of vascular diseases associated with insulin resistance and type-II diabetes associated with obesity.

## Abbreviations

ApoE: Apolipoprotein E

Arg-II: Arginase II

DAF-2DA: 4,5-Diaminofluorescein diacetate

DHE: Dihydroethidium

eNOS: Endothelial nitric oxide synthase

HFD: High fat diet

HUVEC: Human umbilical vein endothelial cells

L-NAME: L-NG-Nitroarginine Methyl Ester

NC: Normal chow

NO: Nitric oxide

p38mapk: p38 mitogen-activated protein kinase

PCR: Polymerase Chain Reaction

rAd: Recombinant adenovirus

WT: Wild type

## Competing interest

The authors declare that they have no competing interests.

## Authors’ contributions

YY, AGR, ZY, and X-FM performed experiments and analyzed data. YY, JPM, ZY, X-FM did manuscript editing. ZY and X-FM carried out the project design, wrote the manuscript. ZY and X-FM are the guarantors of this work and, as such, had full access to all the data in the study and takes responsibility for the integrity of the data and the accuracy of the data analysis. All authors read and approved the final manuscript.

## Authors’ information

YY is a PhD student supported by the Chinese Scholarship Council; AGR is a postdoc; JPM, ZY and X-FM are senior research scientists; ZY and X-FM both are corresponding authors.
